# The Pocket Materia Medica and Therapeutics

**Published:** 1891-07

**Authors:** 


The Pocket Materia Medica and Therapeutics; a Résumé of the Action and Doses of all Officinal and Non-officinal Drugs now in Common Use. By C. Henri Leonard, A. M., M. D., Professor of Medical and Surgical Diseases of

Women and Clinical Gynæcology in the Detroit College of Medicine. Cloth, 12mo, 300 pages; price, postpaid, $1.00. The Illustrated Medical Journal Company, Publishers, Detroit.

This volume, so the preface informs us, has been in preparation for the past four years. The drugs of as late introduction as 1891 are to be found in its pages. The author claims to have incorporated everything of merit, whether officinal or non-officinal, that could be found either in standard works or from many manufacturers' catalogues. The scheme embraces the Pronunciation, Officinal or Non-officinal indication (shown by an *), Genitive, case ending, Common Name, Dose, and Metric Dose. Then the Synonyms, English, French, and German. *If a Plant,* the Part Used, Habitat, Natural Order, and Description of Plant and Flowers, with its Alkaloids, if any. *If a Mineral,* its Chemical Symbol, Atomic Weight, looks, taste, and how found, and its peculiarities. Then the Action and Uses of the Drug, its Antagonists, Incompatibles, Synergists, and Antidotes. Then follow its Officinal and Non-officinal preparations, with their Medium and Maximum Doses, based, so far as possible, upon the last U. S. Dispensatory. Altogether it is a handy volume for either the Physician, Student, or Druggist, and will be frequently appealed to if in one's possession. It is the most complete small book on this subject now issued. It has the same character of compactness and conciseness yet comprehensiveness as the Epitome of *Physical Diagnosis and Urinalysis*, by Dr. John E. Clark, published by the same firm and noticed in the number of this journal for November, 1890, at page 525.

